# Calpain/SHP-1 Interaction by Honokiol Dampening Peritoneal Dissemination of Gastric Cancer in *nu/nu* Mice

**DOI:** 10.1371/journal.pone.0043711

**Published:** 2012-08-24

**Authors:** Shing Hwa Liu, Keh Bin Wang, Keng Hsin Lan, Wen Jane Lee, Hung Chuan Pan, Sheng Mao Wu, Yen Chun Peng, Yi Ching Chen, Chin Chang Shen, Hsu Chen Cheng, Ko Kaung Liao, Meei Ling Sheu

**Affiliations:** 1 Institute of Toxicology, College of Medicine, National Taiwan University, Taipei, Taiwan; 2 Department of Nuclear Medicine, Kuang Tien General Hospital, Taichung, Taiwan; 3 Division of Gastroenterology, Department of Medicine, Taipei Veterans General Hospital, Taipei, Taiwan; 4 Department of Education and Research, Taichung Veterans General Hospital, Taichung, Taiwan; 5 Division of Gastroenterology, Department of Internal Medicine, Taichung Veterans General Hospital, Taichung, Taiwan; 6 Department of Chemistry, National Taiwan University, Taipei, Taiwan; 7 Institute of Nuclear Energy Research, Atomic Energy Council, Taoyuan, Taiwan; 8 Department of Anatomy, Chung Shan Medical University, Taichung, Taiwan; 9 Institute of Biomedical Sciences, National Chung Hsing University, Taichung, Taiwan; 10 Department of life Sciences, National Chung Hsing University, Taichung, Taiwan; Klinikum rechts der Isar der TU München, Germany

## Abstract

**Background:**

Honokiol, a small-molecular weight natural product, has previously been reported to activate apoptosis and inhibit gastric tumorigenesis. Whether honokiol inhibits the angiogenesis and metastasis of gastric cancer cells remains unknown.

**Methodology/Principal Findings:**

We tested the effects of honokiol on angiogenic activity and peritoneal dissemination using *in vivo, ex vivo* and *in vitro* assay systems. The signaling responses in human gastric cancer cells, human umbilical vascular endothelial cells (HUVECs), and isolated tumors were detected and analyzed. In a xenograft gastric tumor mouse model, honokiol significantly inhibited the peritoneal dissemination detected by PET/CT technique. Honokiol also effectively attenuated the angiogenesis detected by chick chorioallantoic membrane assay, mouse matrigel plug assay, rat aortic ring endothelial cell sprouting assay, and endothelial cell tube formation assay. Furthermore, honokiol effectively enhanced signal transducer and activator of transcription (STAT-3) dephosphorylation and inhibited STAT-3 DNA binding activity in human gastric cancer cells and HUVECs, which was correlated with the up-regulation of the activity and protein expression of Src homology 2 (SH2)-containing tyrosine phosphatase-1 (SHP-1). Calpain-II inhibitor and siRNA transfection significantly reversed the honokiol-induced SHP-1 activity. The decreased STAT-3 phosphorylation and increased SHP-1 expression were also shown in isolated peritoneal metastatic tumors. Honokiol was also capable of inhibiting VEGF generation, which could be reversed by SHP-1 siRNA transfection.

**Conclusions/Significance:**

Honokiol increases expression and activity of SPH-1 that further deactivates STAT3 pathway. These findings also suggest that honokiol is a novel and potent inhibitor of angiogenesis and peritoneal dissemination of gastric cancer cells, providing support for the application potential of honokiol in gastric cancer therapy.

## Introduction

The majority of patients (∼60%) with gastric cancer are diagnosed with late stage disease. Gastric cancer is the second most common cause of global cancer mortality in developed nations and exhibits metastatic disease at the time of diagnosis [1]. Surgery and combination chemotherapies for gastric cancer have been shown to confer only modest survival benefits in advanced cases, and nearly 50% of patients still die after recurrence [2], [3]. A major form of recurrence is peritoneal dissemination. Cancer growth and peritoneal metastasis are angiogenesis dependent, a process which involves several angiogenic factors including vascular endothelial growth factor (VEGF), epidermal growth factor (EGF), basic fibroblast growth factor, prostaglandin E2, interleukin-8, chemokine (C-X-C motif) ligand 1, and the matrix metalloproteinase family [4]–[6]. Several molecular signals, such as signal transducer and activator of transcription-3 (STAT-3), nuclear factor-êB, Akt, mitogen-activated protein kinases, cyclooxygenase-2, lipoxygenase, inducible nitric-oxide synthase, tumor necrosis factor and others, have also been shown to be involved in tumor progression and angiogenesis [7]–[9]. However, the cellular and molecular mechanisms of the development, progression, and metastasis of gastric cancer still remain to be clarified.

The Janus-activated kinase (Jak)/STAT signaling pathway plays an important role in the regulation of cell growth, angiogenesis, differentiation, migration, and metastasis [10]. Constitutive activation of STAT pathways, particularly STAT-3, is associated with a wide variety of human malignancies. Persistent STAT-3 phosphorylation has been observed in various human cancers, such as solid tumors of the stomach, colon, liver, prostate, breast, lung, and head and neck as well as blood malignancies [8], [10]. Previous study has shown that phosphorylation of STAT-3 and VEGF protein expression are increased in human gastric cancer tissue, which in turn elevate the angiogenic phenotype and contribute to gastric cancer development and progression [11]. On the other hand, certain phosphatases are known to be tumor suppressors and may play an important role in the inhibition or control of cancer growth [12]–[15]. Protein tyrosine phosphatases (PTPs), including SH2 domain-containing tyrosine phosphatase (SHP)-1 and SHP-2, are able to negatively regulate STAT signaling by the tyrosine dephosphorylation of several components in the related signaling pathways [13]–[15]. The continuous activation of STAT-3 in the tumors might be facilitated at least in part by loss of function of these phosphatases. Calpain II has been shown to play a role in endoplasmic reticulum (ER) stress-regulated tumorigenesis, which is involved in the mechanism of honokiol-inhibited gastric tumorigenesis [16]. In addition, previous study has also indicated that SHP-1 is an endogenous substrate for calpain following A23187-induced platelet activation [17]. Therefore, a calpain/SHP-1-regulated STAT-3 and VEGF pathway may be involved in the angiogenesis, growth, and peritoneal dissemination of gastric cancer cells.

Honokiol, a small-molecular weight natural product, is a major active biphenolic compound of *Magnolia officinalis*, which is known to ameliorate microbial infection, inflammation and gastrointestinal disorders in traditional Asian medicinal systems [18]. Our previous study demonstrated that honokiol inhibits gastric tumorigenesis by activation of 15-lipoxygenase-1 and consequent inhibition of peroxisome proliferator-activated receptor-ã and COX-2-dependent signals [19]. Honokiol has been shown to induce ER stress and trigger calpain-II-mediated glucose-regulated protein-94 cleavage and apoptosis in human gastric cancer cells [16]. It has been demonstrated that VEGF expression could be responsive to nutrient deprivation conditions, which cause ER stress [20], [21]. Moreover, the ER stress activator tunicamycin has been found to markedly prevent microvasculature development, suggesting that this ER stress inducer may have a potential role in breast tumor treatment [21]. The effects of honokiol on ER stress-correlated angiogenesis and gastric tumor metastasis are still unclear. Here, we hypothesized that honokiol inhibits the angiogenesis and peritoneal dissemination of gastric cancer cells through a calpain/SHP-1-regulated STAT-3 and VEGF pathway. The results showed that honokiol markedly inhibited angiogenesis and peritoneal dissemination of gastric cancer cells via a calpain/SHP-1 interaction-activated STAT-3 dephosphorylation and VEGF down-regulation pathway.

## Results

### Honokiol Blocked Peritoneal Metastasis of Gastric Cancer *in vivo*

**Figure 1:**
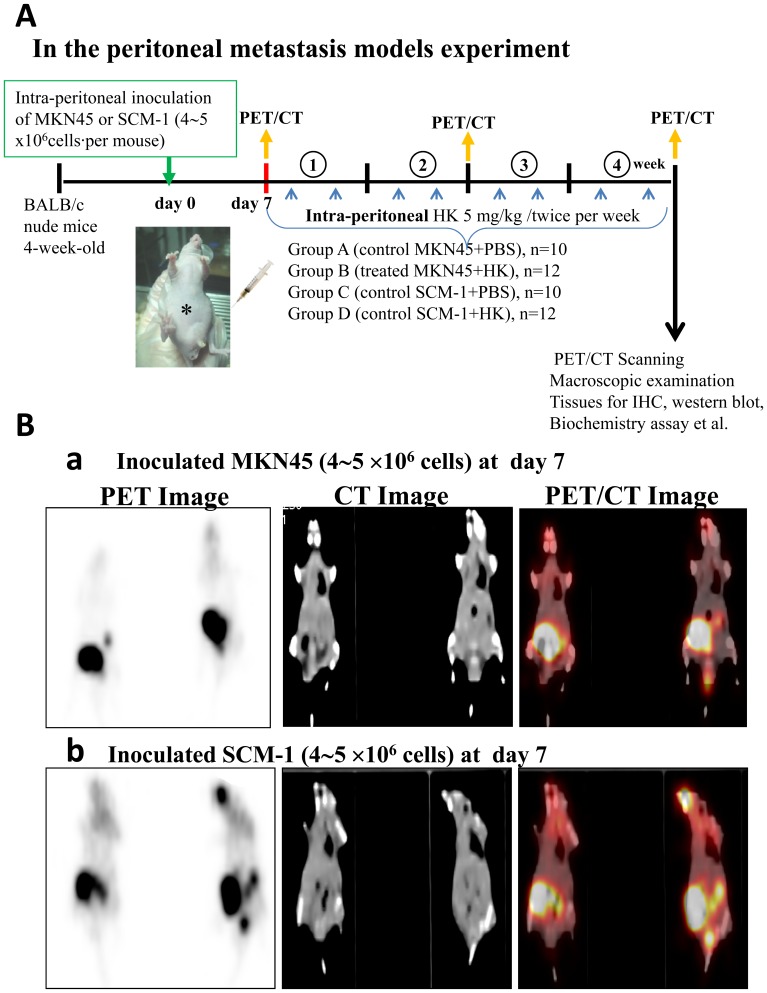
Schematic representation of peritoneal metastasis mouse model with or without honokiol treatment. (A) Four groups of mice were investigated and listed. (B) The metastatic conditions in cancer cell inoculation by PET/CT surveillance and further then for honokiol treatment. The human gastric cancer cells (4∼5×10^6^ cells) MKN45 (a) and SCM-1 (b) were inoculated to mice at day 7.

**Figure 2:**
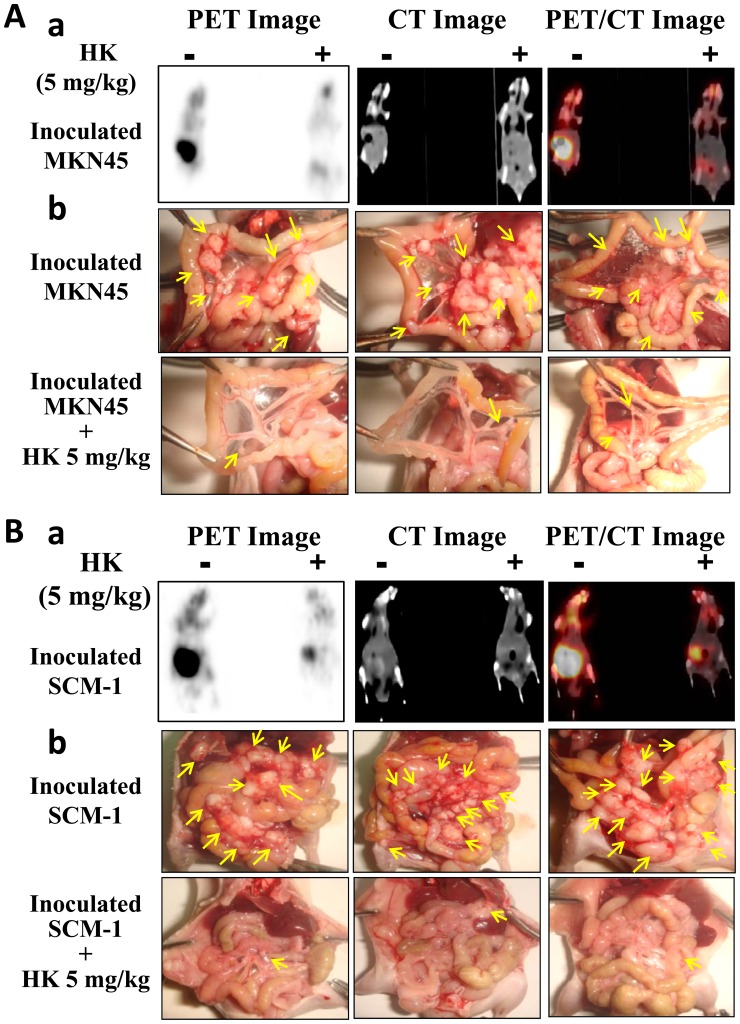
Effect of honokiol on metastasis of gastric tumor cells *in vivo*. [^18^F]-FDG-PET served as a surrogate measure of therapeutic efficacy. Tumors in nude mice were established for 7 days after the intraperitoneal inoculation of gastric cancer cells (MKN45, A; SCM-1, B). Mice were then injected intraperitoneally with honokiol (5 mg/kg/twice per week). Twenty-eight days after honokiol treatment, FDG-PET/CT images of mice were taken, and then mice were sacrificed for macroscopic examination of the distribution of disseminated metastasis. Representative FDG-PET/CT images of animals inoculated with MKN45 (A-a) or SCM-1 (B-a) gastric cancer cells with or without honokiol (HK) treatment are shown. HK (5 mg/kg) was administered by intraperitoneal injection. The maximum intensity projection of typical representative nude mice (left, tumor control; right, HK treatment) is shown. Moreover, many metastatic nodules were found in the mesentery of the control mice inoculated with MKN45 (A-b) or SCM-1 (B-b) cells. In contrast, peritoneal metastasis was observed sporadically in honokiol-treated mice.

**Figure 3:**
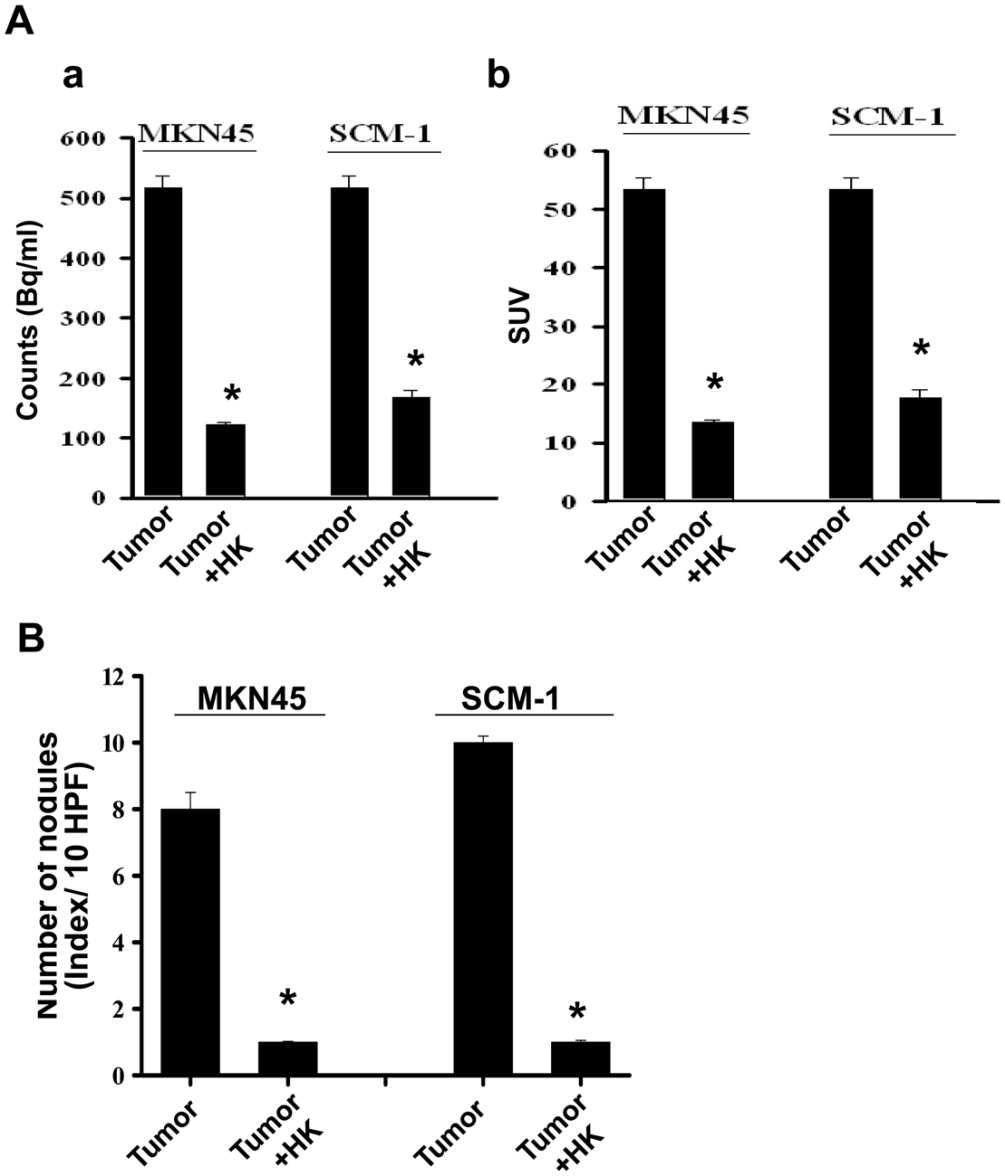
Quantification of honokiol-inhibited peritoneal metastasis of gastric tumor cells. Mice were inoculated with human gastric cancer cells (MKN45 and SCM-1). [^18^F]-FDG-PET/CT imaging was performed and analyzed. (A) Quantifications of estimated radioactivity (Bq/ml, a) and specific uptake values (SUV, b) were calculated. SUV is used as an index to determine if a hotspot is significant. (B) Photomacrographs of metastatic peritoneal nodules are shown. All data are presented as mean ± SEM (n = 6–8). *p<0.05 as compared with control.

Cancer is frequently characterized by the increased uptake of [^18^F]-fluoro-2-deoxy-D-glucose (FDG), and [^18^F]-FDG/PET can serve as a surrogate measure of therapeutic efficacy. The functional possibility of small animal imaging using a clinical PET/CT scanner with FDG was evaluated. Four groups of mice for peritoneal metastasis experiments were investigated. A statement/picture of the metastasis situation was shown in Figure 1. We utilized [^18^F]-FDG-PET/CT to detect peritoneal metastases in mice inoculated with human gastric cancer cells (MKN45 or SCM-1) with or without honokiol treatment. As shown in Figure 2, the maximum intensity projection was generated from typical representative mice. The peritoneal metastasis was marked in the control mice (left panel), and it was effectively reversed by the honokiol treatment (right panel). These images clearly showed that noninvasive [^18^F]-FDG uptakes in peritoneal metastatic tumors of control mice were much higher than those in the honokiol-treated mice. The quantification of intensity is shown in Figure 3A. Intraperitoneal injection of honokiol (5 mg/kg, twice/weekly) significantly reduced the estimated radioactivity counts and specific uptake values (SUV) determined by FDG-PET/CT in mice inoculated with gastric cancer cells (Fig. 3). Moreover, many metastatic nodules were found in the peritoneal cavity (mesentery) of the control mice inoculated with MKN45 and SCM-1 (Fig. 2) cells. In contrast, peritoneal tumor nodules were observed sporadically in mice treated with honokiol. Quantification of nodules per field is shown in Figure 3B.

### Honokiol Inhibited Angiogenesis *in vivo*, *ex vivo*, and *in vitro*

**Figure 4:**
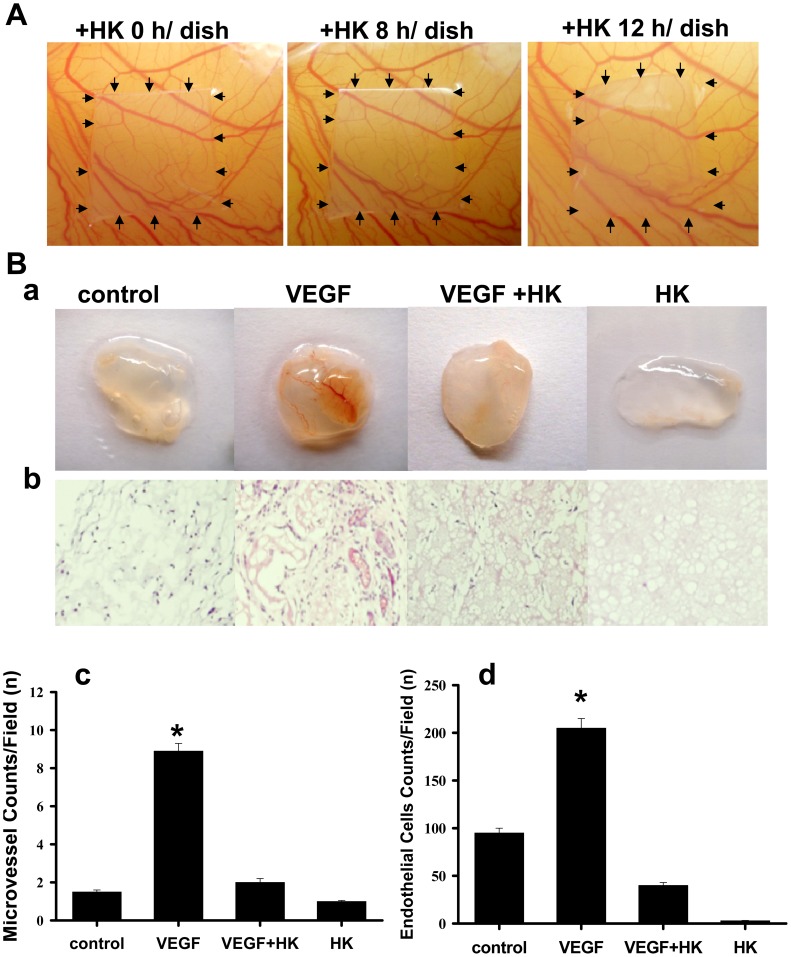
Effect of honokiol on angiogenesis. (A) Chorioallantoic membrane assay (*CAM* assay) was performed. Neovascular formation entrapped in type I collagen gels with or without honokiol (HK, 20 µM) treatment was examined. After 24-h incubation, the area around the loaded disk was photographed. (B) Matrigel (0.6 ml) was implanted subcutaneously in nude mice with or without honokiol (10 µg/ml) or VEGF (100 ng/ml). The injected matrigel rapidly formed a single solid gel plug. After 21 days, mice were sacrificed and matrigel plugs were excised. Representative matrigel plugs (a) and IHC for CD31 staining of sections from matrigel plugs (b) are shown. Quantifications of vessel numbers (c) and endothelial cells (d) in matrigel plug sections (counts/field) were calculated. The numbers in each case are the average from five different slides and five regions per slide. All data are presented as mean ± SEM (n = 8–10). *p<0.05 as compared with control.

Endothelial cells are critical for the angiogenic process, which is necessary for tumor growth and metastasis. We next investigated the angiogenic effect of honokiol using the chick chorioallantoic membrane assay (*CAM* assay), matrigel plug assay, aortic ring sprouting assay, and endothelial cell tube formation. As shown in Figure 4A, honokiol effectively inhibited the neo-vascular formation in the *CAM* assay without any visible effect on the pre-existing blood vessels. Quantitative analysis revealed that honokiol caused a 2.5-fold decrease in the number of newly formed blood vessels as compared with that of medium control.

In the matrigel plug assay, matrigel containing VEGF (100 ng/ml gel) with or without honokiol (10 µg/ml) was subcutaneously implanted into nude mice. After 21 days of implantation, the formed matrigel plugs were excised and photographed. Plugs with VEGF alone were markedly streaky, vascular and red in color. Plugs containing VEGF and honokiol were pallid, indicating no or less blood vessel formation (Fig. 4B-a). We also examined the vessel density and vessel morphology in the plug sections by H & E staining and immunohistochemical staining with an antibody against CD31, an endothelial cell marker. The vascularization in the honokiol+VEGF group was significantly reduced as compared with the VEGF alone group (Fig. 4B-b). Quantification of the vascularization by counting the vessels and presented endothelial cells revealed that the vascular density in the honokiol-treated group was significantly decreased (Figs. 4B-c and 4B-d).

**Figure 5:**
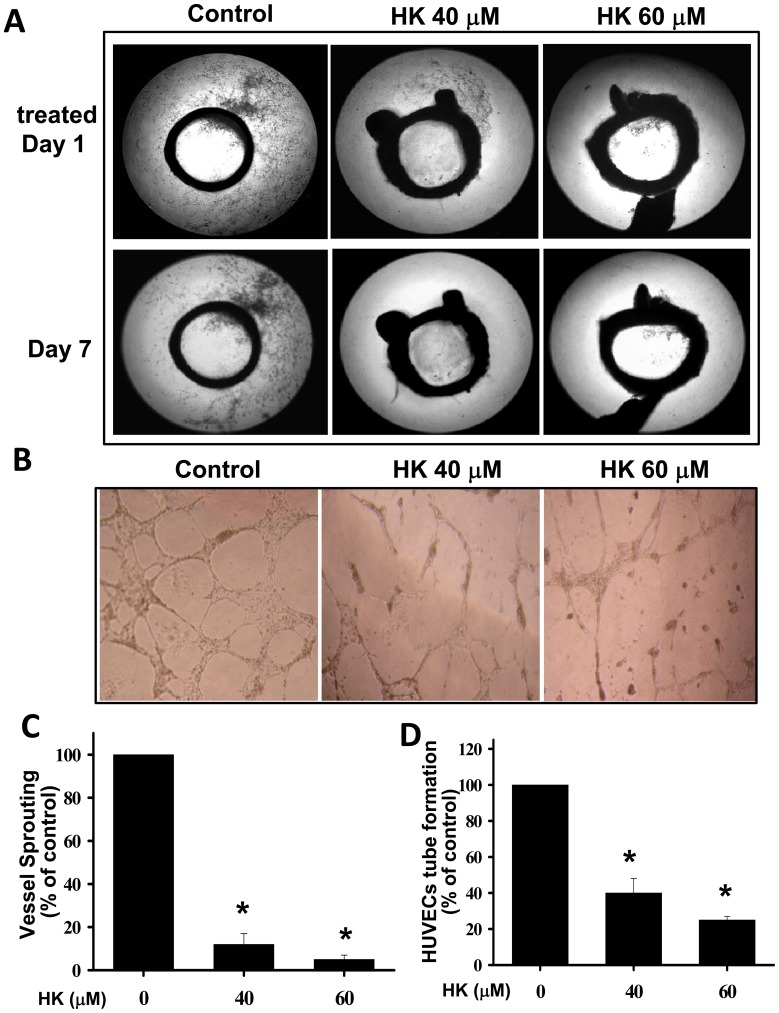
Effects of honokiol on endothelial cell sprouting and endothelial cell tube formation. (A) Honokiol inhibited endothelial cell sprouting in an aortic ring assay. Aortas were harvested from 6-week-old Sprague-Dawley rats and cut into 1-mm slices, which were then placed in 12-well plates containing matrigel. The rings were photographed and analyzed. The endothelial cell sprouting was abundant in the control aortic rings (left panels), but not in the rings treated with honokiol (HK; middle panels, 40 µM; right panels, 60 µM). (B) Honokiol suppressed the endothelial cell tube formation. HUVECs were seeded into matrigel-coated 96-well plates. Cells were treated with honokiol (40 and 60 µM) for 12–18 h. The tube formation images were captured in an inverted photomicroscope, and tube formations were scored. (C) The degree of microvessel sprouting from aortic rings was scored from 0 (least positive) to 4 (most positive). (D) Quantification of HUVEC tube formation is shown. All data are presented as mean ± SEM (n = 8–10). *p<0.05 as compared with control.

Next, endothelial cells were induced to sprout from the isolated rat aortic rings in the presence of matrigel and ECGM (endothelial cell growth medium) containing angiogenic cytokines, such as VEGF and basic fibroblast growth factor. Extensive endothelial cell outgrowth from rat aorta ring explants was observed in the control group (Fig. 5A). Honokiol treatment resulted in a significant (∼five-fold) reduction of endothelial outgrowth and sprouting from aortic rings (Figs. 5A and 5C). Furthermore, the morphological differentiation of endothelial cell tube formation was investigated using a two-dimensional matrigel method. As shown in Figure 5B, seeding of HUVECs in matrigel led to vascular tube-like structure formation. Honokiol effectively inhibited the endothelial cell tube formation by reducing the tube-like structure in length and width (Figs. 5B and 5D).

### Honokiol Abolished STAT-3 Signaling in Human Gastric Cancer Cells, HUVECs, and Tumors

**Figure 6:**
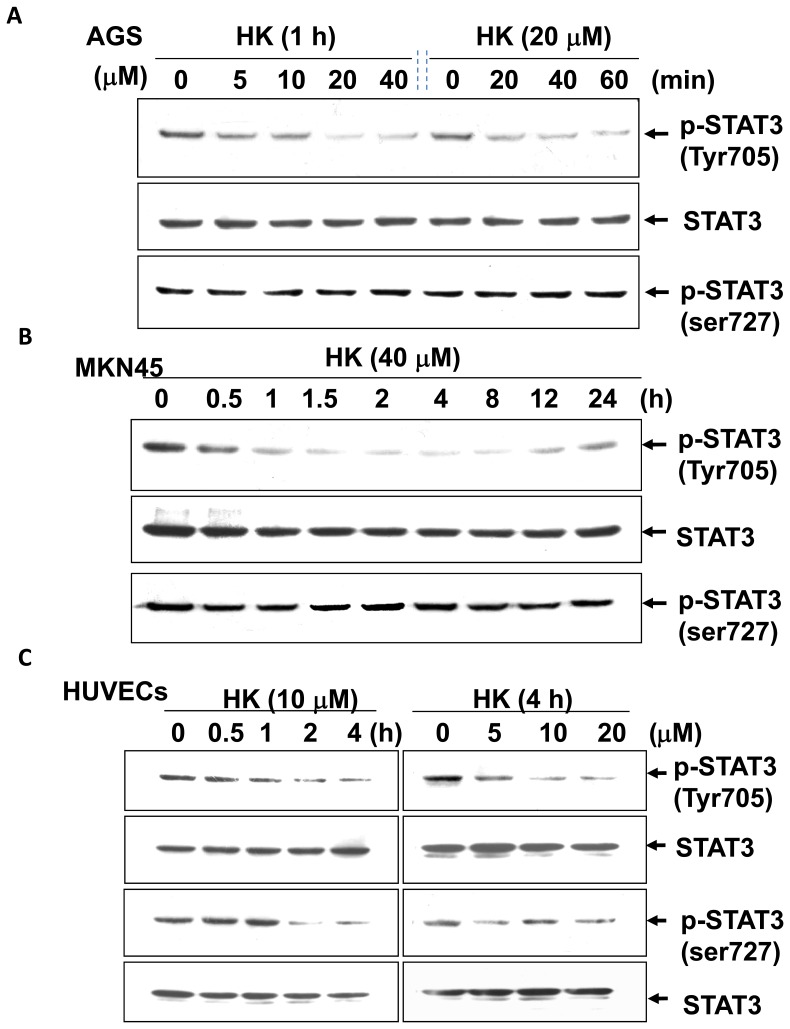
Effect of honokiol on STAT-3 phosphorylation in gastric cancer cells and HUVECs. AGS (A) and MKN45 (B) quiescent human gastric cancer cells and HUVECs (C) were treated with and without honokiol (HK, 5–40 µM) for the indicated times. Whole cell lysates were prepared and analyzed by Western blotting for detection of phosphorylated STAT-3 (pSTAT-3, Tyr705). The blottings were reprobed with anti-STAT-3 antibodies for normalization. The results are representative of at least five independent experiments.

**Figure 7:**
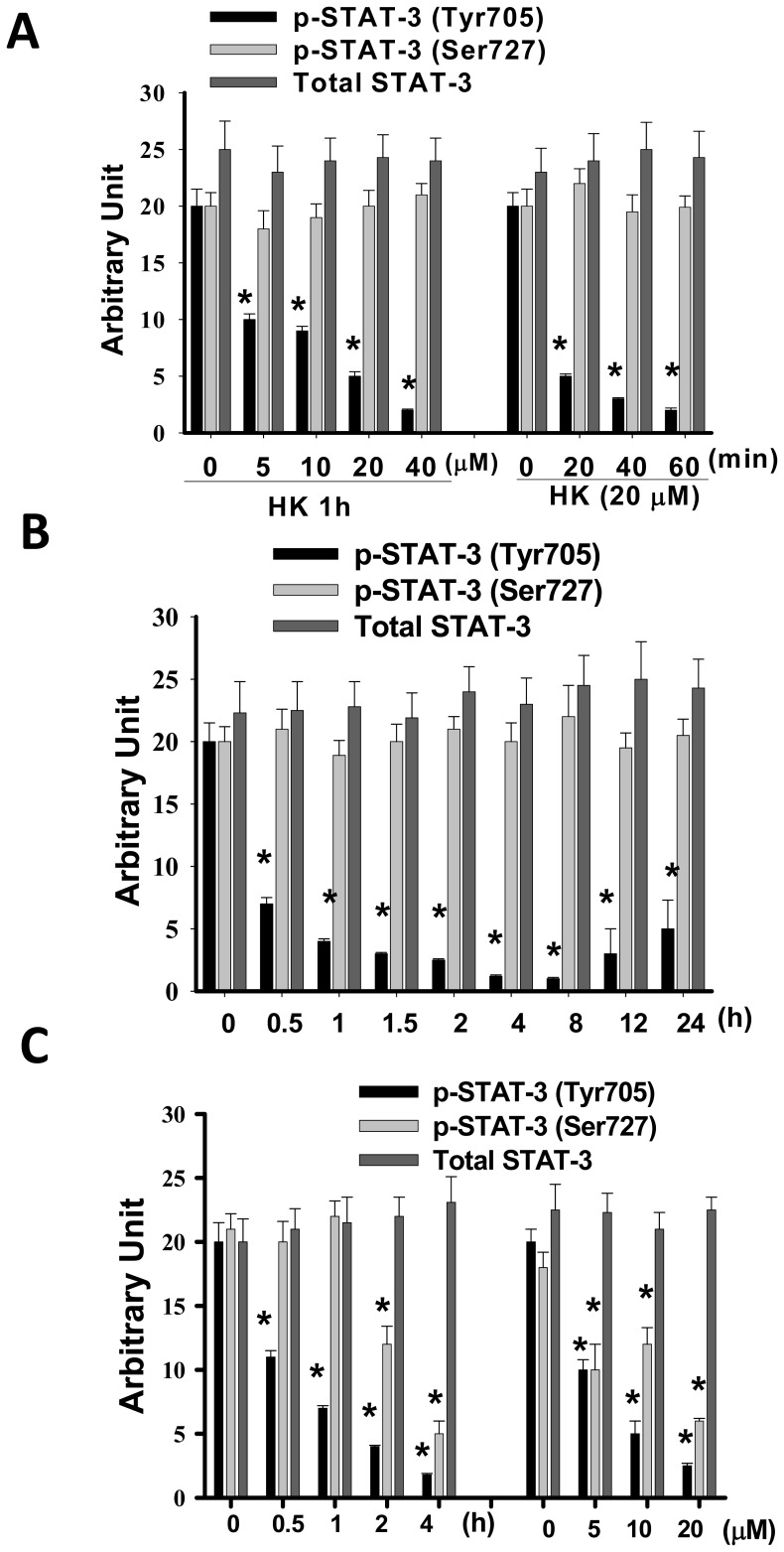
Quantification of honokiol-inhibited STAT-3 phosphorylation in gastric cancer cells and HUVECs. AGS (A) and MKN45 (B) quiescent human gastric cancer cells and HUVECs (C) were treated with and without honokiol (HK, 5–40 µM) for the indicated times. Whole cell lysates were prepared and analyzed by Western blotting for detection of phosphorylated STAT-3 (pSTAT-3, Tyr705). The blottings were reprobed with anti-STAT-3 antibodies for normalization. All data are presented as mean ± SEM of five independent experiments. *p<0.05 as compared with control.

**Figure 8:**
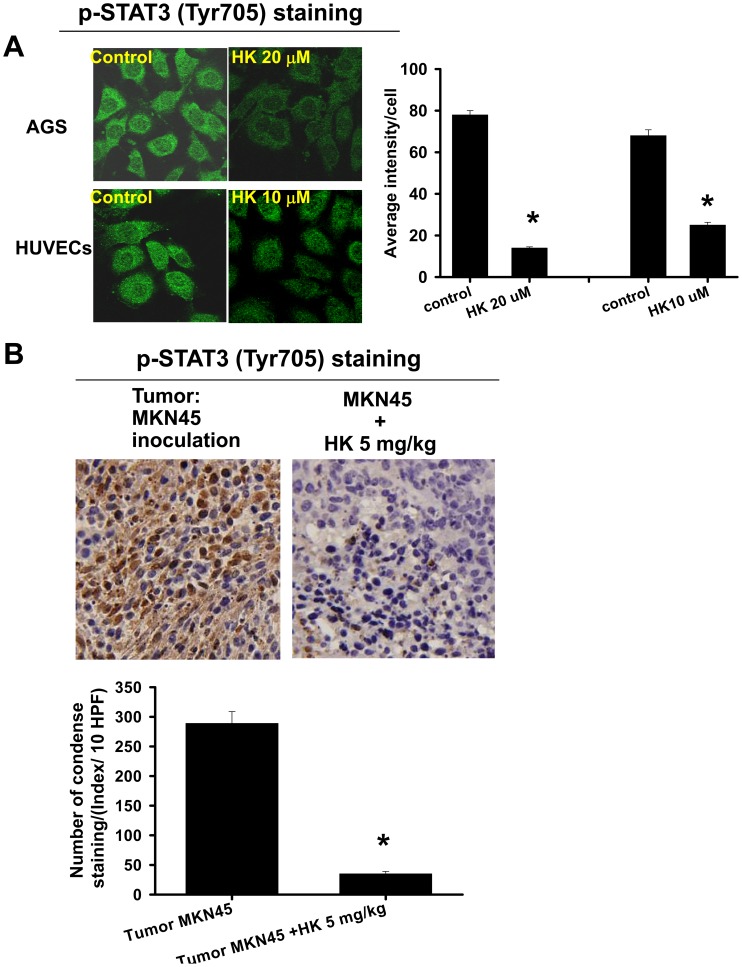
Effects of honokiol on the phosphorylation of STAT-3 in gastric cancer cells, HUVECs, and tumors. (A) The p-STAT-3 (Tyr705) expressions in gastric cancer cells (AGS) and HUVECs treated with honokiol (HK, 10 and 20 µM) for 1 h were detected by a confocal microscope. (B) Tumors in nude mice were established for 7 days after the intraperitoneal inoculation of MKN45 cells. Mice were then injected intraperitoneally with honokiol (5 mg/kg/twice per week) for 28 days. The p-STAT-3 (Tyr705) expressions in metastatic tumors isolated from mice with or without HK treatment are shown. The p-STAT-3 (Tyr705) protein expressions, stained dark brown, were detected by immunohistochemistry. All data are presented as mean ± SEM of five independent experiments. *p<0.05 as compared with control.

Previous studies have shown a strong correlation between activated STAT-3 and angiogenic phenotype [11]. We further elucidated the effect of honokiol on the phosphorylation of STAT-3 in human gastric cancer cells (MKN45 and AGS) and HUVECs. As shown in Figure 6, honokiol reduced the tyrosine (Tyr705) phosphorylation of STAT-3 in a time-dependent manner with about a two-fold decrease at 1–2 h in AGS cells (Fig. 6A) and a 3 to 10-fold decrease at 0.5–24 h in MKN45 cells (Fig. 6B). Surprisingly, honokiol induced STAT-3 dephosphorylation of Tyr705, but not Ser727, residues in these human gastric cancer cells. However, honokiol simultaneously inhibited both Tyr705 and Ser727 phosphorylation of STAT-3 in HUVECs (Fig. 6C). Quantitative analysis of protein bands in the Western blot using Image-Pro Plus software is shown in Figure 7. The results of analysis with confocal microscope also showed that honokiol abolished the tyrosine (Tyr705) phosphorylation of STAT-3 in AGS cells and HUVECs (Fig. 8A).

**Figure 9:**
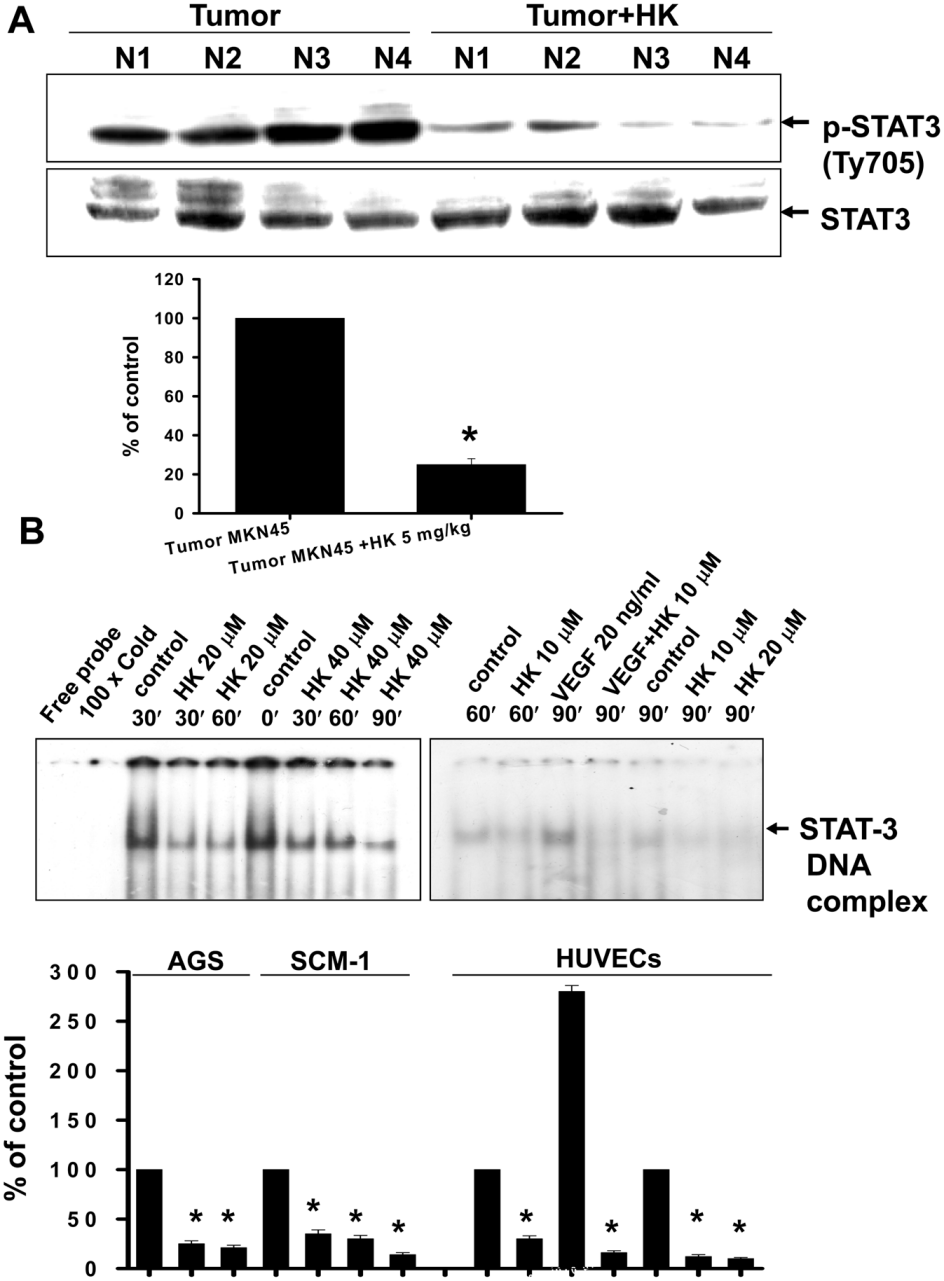
Effects of honokiol on p-STAT-3 expression in tumors and STAT-3 DNA binding activity in gastric cancer cells and HUVECs. (A) Western blotting for detection of p-STAT-3 (Tyr705) proteins in metastatic tumors isolated from mice with or without honokiol (HK) treatment is shown. (B) EMSA was performed for detection of STAT-3 DNA binding activity in AGS, SCM-1 (left panel), and HUVECs (right panel) with or without honokiol treatment. Cells were treated with honokiol for various time courses as indicated. VEGF (20 ng/ml) enhanced the STAT-3 DNA binding activity in HUVECs, and this enhancement was reversed by honokiol (10 µM). All data are presented as mean ± SEM of five independent experiments. *p<0.05 as compared with control.

We further examined the STAT-3 phosphorylation in peritoneal metastatic tumors isolated from nude mice inoculated with MKN45 cells with or without honokiol (5 mg/kg) treatment. Immunohistochemical analysis showed that p-STAT-3 overexpression and accumulation in the tumor region, including nuclei and cytoplasm, was significantly reversed by honokiol treatment (Fig. 8B). In Western blotting analysis, honokiol markedly decreased the accumulation of p-STAT3 in tumors as compared with vehicle control (Fig. 9A). The expression of constitutive STAT3 was not affected. Moreover, the DNA binding activity of STAT-3 was further confirmed using EMSA. As shown in Figure 9B, honokiol markedly inhibited the increased DNA binding activity of STAT-3 in human gastric cancer cells. Honokiol also inhibited the VEGF-increased STAT-3 DNA binding activity in HUVECs (Fig. 9B).

### Honokiol Induced SHP-1-regulated STAT-3 Dephosphorylation in Gastric Cancer Cells, Endothelial Cells, and Tumors

**Figure 10:**
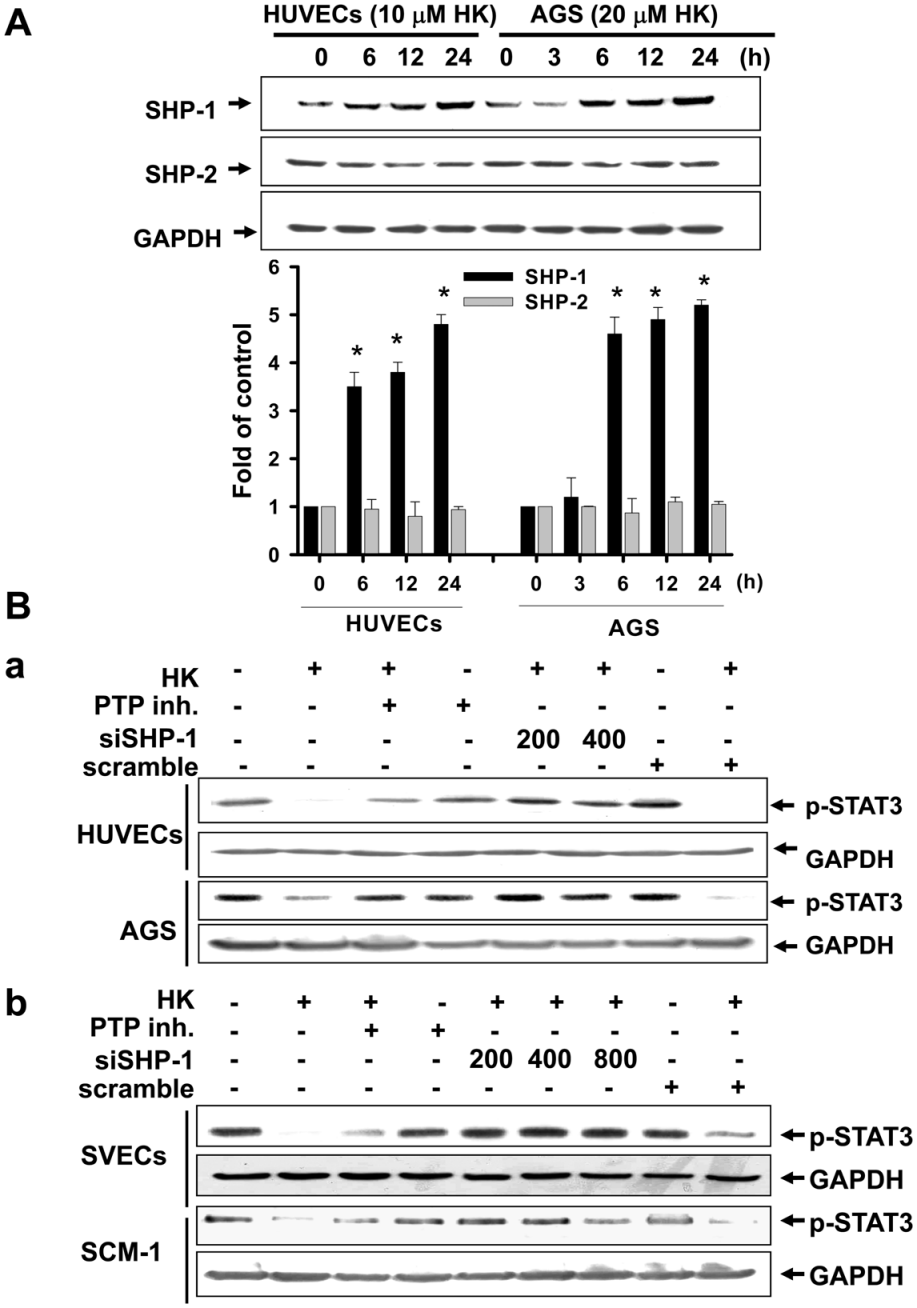
Honokiol enhanced phosphatase SHP-1 protein expression and the interaction with STAT-3 in gastric cancer cells and HUVECs. Cells were treated with honokiol (10 and 20 µM) for various time courses as indicated. (A) SHP-1 and SHP-2 protein levels were detected by Western blot analysis in cells with or without honokiol treatment. (B) The phosphorylation of STAT-3 in gastric cancer cells (AGS and SCM-1) and endothelial cells (HUVECs and SVECs) with or without honokiol (10 µM in HUVECs, SVECs, and SCM-1; 20 µM in AGS) treatment for 24 h in the presence or absence of a phosphatase inhibitor (PTP inhibitor II, 20 µM) or SHP-1 siRNA transfection was detected. In A, data are presented as mean ± SEM of five independent experiments. *p<0.05 as compared with control. In B, the results shown are representative of at least four independent experiments.

**Figure 11:**
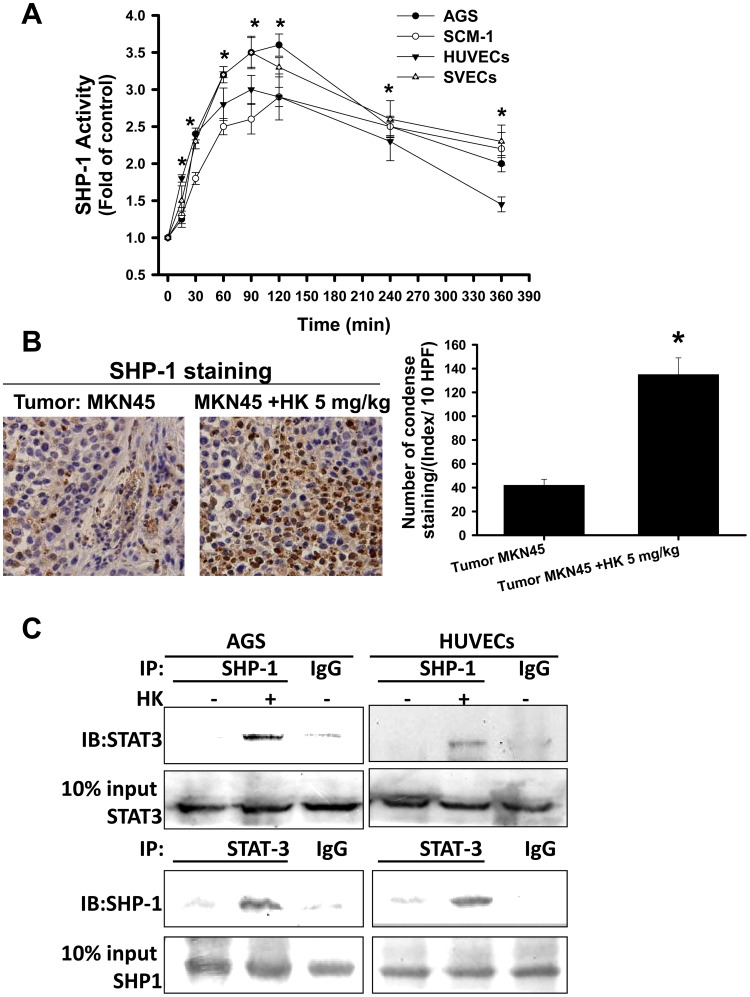
Honokiol enhanced the phosphatase SHP-1 activity and the interaction with STAT-3 in gastric cancer cells and endothelial cells and the SHP-1 protein expression in tumors. Cells were treated with honokiol (HK) for various time courses as indicated. (A) Cells were treated with honokiol (HUVECs, 20 µM; SVECs, 20 µM; AGS, 20 µM; SCM-1, 40 µM) at the indicated times, and then the SHP-1 activities were measured. Data are presented as mean±SEM (n = 7). *P<0.05 as compared with control. (B) Immunohistochemistry for SHP-1 expression in metastatic tumors isolated from mice with or without HK treatment is shown. The sections were stained with anti-SHP-1 antibody. (C) Interaction of STAT-3 and SHP-1 was detected in AGS cells or HUVECs. Immunoprecipitated proteins were collected and subjected to SDS-PAGE and immunoblotting with anti-STAT-3 or anti-SHP-1 antibodies. In A and B, data are presented as mean ± SEM (n = 5). *p<0.05 as compared with control. In C, the results shown are representative of at least four independent experiments.

SHP-1 is a non-transmembrane PTP [12]. We next examined whether honokiol can regulate the expression and activity of SHP-1. As shown in Figure 10A, honokiol enhanced the protein expression of SHP-1, but not SHP-2, in HUVECs and AGS cells in a time-dependent manner. Pharmacological PTP inhibitor and SHP-1 siRNA transfection effectively abolished the honokiol-induced STAT-3 dephosphorylation in HUVECs and AGS cells (Fig. 10B-a). Similar results in SCM-1 cells and SV-40 immortalized mouse microvascular endothelial cells (SVECs) treated with honokiol are shown in Figure 10B-b. We next examined whether endogenous SHP-1 is modulated by honokiol. Honokiol was capable of evoking the SHP-1 activity in gastric cancer cells, HUVECs and SVECs in a time-dependent manner (Fig. 11A). Honokiol also enhanced the SHP-1 protein expression in peritoneal metastatic tumors isolated from MKN45-inoculated mice as revealed by immunohistochemical analysis (Fig. 11B). We further investigated the interaction between SHP-1 and STAT-3 using the methods of co-immunoprecipitation and Western blotting. As shown in Figure 11C, SHP-1 was specifically associated with STAT-3 in AGS cells and HUVECs in the presence of honokiol as compared with IgG control.

### Honokiol Altered ER Morphology, Induced Calpain-II-regulated SHP-1-induced STAT-3 Dephosphorylation, and Decreased VEGF Generation

**Figure 12:**
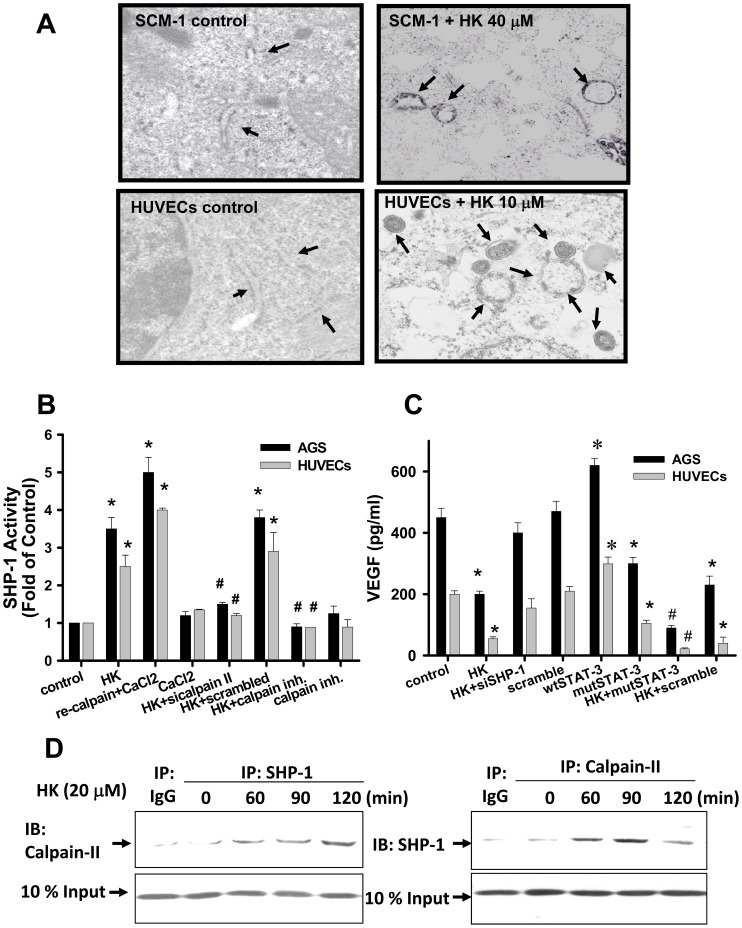
Effects of honokiol on ER structure, SHP-1 activity, and VEGF generation. (A) Cells were treated with honokiol (HUVECs, 10 µM; SCM-1, 40 µM) for 18 h. Cells were collected and visualized by electron microscopy as described in “Materials and Methods”. Arrows indicate dilated ER. Original magnification: 9800x. (B) Cells were treated with or without honokiol (HUVECs, 20 µM; AGS, 20 µM) in the presence or absence of recombinant protein of calpain II (re-calpain) or calpain II siRNA transfection, and then cells were analyzed for SHP-1 activity. (C) Cells were transfected with wild type STAT-3 (wt) and mutant STAT-3 (mut) plasmids or SHP-1 siRNA, and then the VEGF generation was determined. (D) Interaction of calpain and SHP-1 was detected in AGS cells. Immunoprecipitated proteins were collected and subjected to SDS-PAGE and immunoblotting with anti-calpain-II or anti-SHP-1 antibodies. In A and D, results shown are representative of at least 4 independent experiments. In B and C, data are presented as mean ± SEM (n≥4). *P<0.05 as compared with control. #P<0.05 as compared with honokiol alone.

Previous studies have suggested that ER stress plays an important role in angiogenesis [20], [21]. We next examined the effect of honokiol on ER microstructure by transmission electron microscopy. As shown in Figure 12A, ER dilation and fragmentation were exhibited in honokiol-treated SCM-1 gastric cancer cells and HUVECs.

We also wanted to determine whether calpain-II activation is required for SHP-1 activity in honokiol-treated cells. Pharmacological calpain inhibitor and calpain-II siRNA transfection effectively reduced honokiol-enhanced SHP-1 activity in AGS cells and HUVECs (Fig. 12B). Cells treated with recombinant calpain-II, as a positive control, showed a 4 to 5-fold increase in SHP-1 activity (Fig. 12B). VEGF is known to contain the STAT-3 promoter binding site. We next examined whether honokiol can regulate VEGF expression. Honokiol significantly reduced the VEGF generation in AGS cells and HUVECs, and this effect was reversed by SHP-1 siRNA transfection (Fig. 12C). In addition, transfection of mutant-STAT-3 plasmid into cells also inhibited the VEGF generation. The combination of honokiol with mutant-STAT-3 plasmid transfection in cells synergistically reduced the VEGF production as compared with either treatment alone (Fig. 12C). To further confirm the interaction between calpain-II and SHP-1, co-immunoprecipitation and Western blotting were performed in gastric cancer cells. As shown in Figure 12D, calpain-II was specifically associated with SHP-1 in AGS cells in the presence of honokiol (20 µM) as compared with IgG control. Similarly, the honokiol-induced calpain/SHP-1 interaction was also found in HUVECs (data not shown).

## Discussion

Honokiol is a major component of *Magnolia officinalis*, which is a transitional medicine in Asia [18]. Honokiol possesses anti-oxidative and anti-inflammatory effects *in vitro* and *in vivo* [22]–[25]. Honokiol has been shown to exhibit potent anti-proliferative activity against endothelial cells *in vitro* and anti-tumor effects against angiosarcoma in nude mice [26]. In this study, we demonstrated for the first time that honokiol is a potent inhibitor of angiogenesis and peritoneal metastasis of gastric cancer. Our research has focused on the effects and possible mechanisms of honokiol on peritoneal metastasis and angiogenesis using PET/CT, *CAM* assay, matrigel plug assay, aortic ring endothelial cell sprouting assay, endothelial cell tube formation assay, and *in vitro* cell experiments. The results showed that honokiol can suppress angiogenesis and peritoneal dissemination through a calpain-II-activated SHP-1 activation signaling cascade. Activation of phosphatase SHP-1 by honokiol further led to the down-regulation of STAT-3 activation and VEGF production, resulting in the inhibition of angiogenesis and peritoneal metastasis. However, the inhibition of angiogenesis and inhibition of metastatic potential of cancer cells by honokiol may be the separate aspects, one being an effect of honokiol on angiogenesis directly and another effect of honokiol on cancer cells.

During the past several years, a number of studies have indicated that constitutive activation of the STAT family, especially STAT-3, is associated with cell proliferation, angiogenesis and metastasis [10], [11], [27]–[30]. Increased activation of STAT-3 expression has been found in various tumor-derived cells and samples from human cancer tissues. Deregulated STAT-3 activation has been suggested to play an important role in overexpression of VEGF and increased angiogenic phenotypes in gastric cancer, which may contribute to gastric cancer development and progression [11]. In the present study, we found that honokiol can effectively inhibit the phosphorylation of STAT-3 in HUVECs (both Tyr705 and Serine727 sites), human gastric cancer cells (Tyr705 site), and peritoneal metastatic tumors (Tyr705 site). As the oncogenic activation of tyrosine kinases is a common feature in cancers, we focused on the role of Tyr705 phosphorylation of STAT-3.

SHP-1 possesses a potential tumor suppressor function and is a negative regulator of the JAK/STAT signaling pathway [15]. It has also been shown that SHP-1 phosphatase can bind to the phosphorylated Y1173 domain, which leads to EGFR dephosphorylation [31]. Y429 in the cytoplasmic domain of the erythropoietin receptor has been suggested to be the binding site for protein tyrosine phosphatase SH-PTP1, which may play a major role in terminating proliferative signals [32]. SHP-1 has also been shown to be an antagonist of growth-factor signaling in epithelial and hematopoietic cells [15]. SHP-1 was particularly highlighted as a potential physiological antagonist of VEGFR signaling [33]. In endothelial cells, SHP-1 is physically associated with VEGFR2 and is also required for both TNFα-mediated and tissue-inhibitor of metalloproteinases (TIMP)-mediated inhibition of angiogenesis [31]. Thus, SHP-1 activation may have important consequences for the regulation of proliferation and angiogenesis. In this study, we found that honokiol specifically enhanced the expression of SHP-1, but not SHP-2, in gastric cancer cells, endothelial cells, and peritoneal metastatic tumors. These findings suggest that honokiol may inhibit endothelial cell angiogenesis and cancer cell metastasis, which may be through a SHP-1-induced STAT-3 down-regulation pathway.

Previous studies have proposed an important role for ER stress in the molecular mechanism of angiogenesis and cancer cell growth [16], [20], [21]. Koyama and colleagues have also suggested that ER stress-induced calcium disruption may be involved in the induction of amyloid β accumulation and angiogenic factor expression in the retinal pigment epithelium [34]. A recent study has shown that ER stress-regulated IRE-1α signaling possesses an essential function in placental development and embryonic viability, highlighting the relationship of ER stress, and angiogenesis in the placenta during pregnancy [35]. Kim and colleagues have shown that both SHP-1 and SHP-2 are endogenous substrates for calpain, which is involved in the context of *Entamoeba histolytica*-induced host cell death [36]. Notably, calpains become active when the intracellular calcium ([Ca^2+^]i) concentration is elevated; m-calpain (calpain II) requires [Ca^2+^]i at a millimolar range, but micromolar concentrations of [Ca^2+^]i are enough for the activation of μ-calpain (calpain I) [37], [38]. It has been shown that a high level of [Ca^2+^]i hinders protein tyrosine kinase activity as a result of calpain activation during stimulation by ionophore in platelets [39]. Honokiol was capable of inducing [Ca^2+^]i mobilization in neuronal cells [40]. Recently, tunicamycin has been reported to inhibit angiogenesis *in vivo*. Our previous study has shown that honokiol induces human gastric cancer cell apoptosis and inhibits tumorigenesis through a calpain-mediated glucose-regulated protein-94 cleavage [16]. In the present study, we straightforwardly demonstrated that the ER were dilated and fragmented in honokiol-treated gastric cancer cells and HUVECs. Moreover, pharmacological inhibitor of calpain and calpain-II siRNA transfection effectively reduced honokiol-enhanced SHP-1 activity in gastric cancer cells and HUVECs. Notably, our results for the first time demonstrated that calpain/SHP-1 interaction is directly triggered by honokiol inhibition of STAT-3 phosphorylation in gastric cancer cells. These results indicate that honokiol can induce ER stress and trigger the calpain-II-activated SHP-1 activity in gastric cancer cells and endothelial cells.

**Figure 13:**
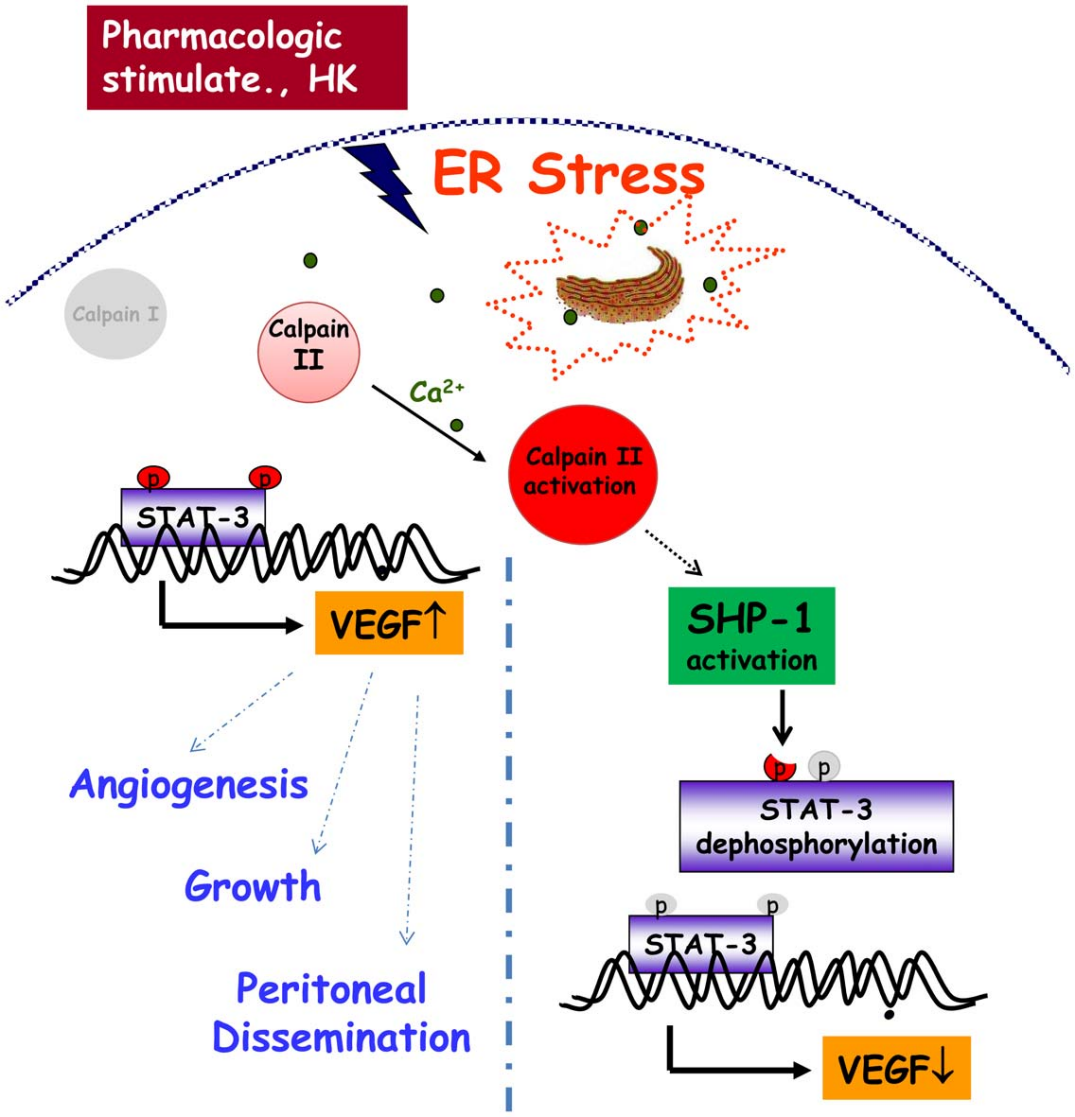
A proposed mechanism for honokiol up-regulation of endoplasmic reticulum stress, which reduced angiogenesis, growth, and peritoneal dissemination in gastric cancer cells through the calpain II/SHP-1/STAT-3 pathway. Honokiol induced the SHP-1 activity and resultant cell STAT-3 dephosphorylation through endoplasmic reticulum stress activation and calpain-II regulation.

In conclusion, the results of the present study provide *in vivo, ex vivo* and *in vitro* evidence for inhibition of angiogenesis and peritoneal dissemination of gastric cancer cells by honokiol and a molecular basis for its effects. These data highlight the involvement of ER calpain and protein tyrosine phosphatase SHP-1 signaling in honokiol-inhibited STAT-3 activation and VEGF production in gastric cancer cells and endothelial cells (Fig. 13). These findings also suggest that the down-regulation of STAT-3 and VEGF signaling induced by calpain/SHP-1 may be a promising therapeutic strategy for gastric cancer treatment.

## Materials and Methods

### Cells and Culture

Human gastric cancer cell lines including a Caucasian cell line (AGS, moderately/poorly (mixed) differentiated adenocarcinoma cells) and Asian cell lines (MKN45 and SCM-1, undifferentiated adenocarcinoma cells) were obtained from the cell bank of Taipei Veterans General Hospital (Taiwan). Cells were maintained in RPMI1640 medium containing 10% FCS and streptomycin/penicillin in a humidified 5% CO_2_ atmosphere.

HUVECs were obtained from umbilical cord veins by collagenase treatment as described previously [41]. This study conforms to the principles for use of human tissues outlined in the Declaration of Helsinki. The study protocol was approved by the Ethics Review Board of Taichung Veterans General Hospital, Taichung, Taiwan (approved document TCVGH-C0062-1). HUVECs at the third passage were used. The efficiency of transfection (>80%) by Lipofectin reagent (Invitrogen) was determined at the third passage. In some experiments, SV-40 immortalized mouse microvascular endothelial cells (SVECs) cultured in Dulbecco’s modified Eagle’s medium (DMEM) supplemented with 10% fetal bovine serum (FBS) and 1% penicillin/streptomycin. SVEC cell line was kindly gifted by Dr. KH Lan (Cancer Center, Taipei Veterans General Hospital, Taiwan).

Honokiol was obtained from Wako Chemical Company (Osaka, Japan), and it was determined to be at least 99% pure by HPLC. Different concentrations of honokiol were used in this study depending on the drug sensitivity indicated by IC50 values (20 µM in AGS, 40 µM in MKN45 and SCM-1). MKN45 and SCM-1 cells, but not AGS cells, have been shown to effectively induce tumors in a xenograft tumor mouse model.

### Animals

All animal care and experimental procedures were approved and conducted by the Committee for Animal Experiments, National Chung Hsing University, Taichung, Taiwan (approved document NCHU-100-26). Four- to six-week-old male BALB/c nude mice were purchased from NLAC (Taipei, Taiwan). The mice were bred and maintained under specific pathogen-free conditions, provided with sterilized food and water *ad libitum* and housed in a barrier facility with a 12 h light/dark cycle.

### Xenograft Tumor Mouse Model

To evaluate the peritoneal metastasis, cultured human gastric cancer cells (4∼5×10^6^ cells) were inoculated into the peritoneal cavity of BALB/c nude mice [42]. Peritoneal tumors in nude mice were established for 7 days after the injection of gastric cancer cells, in which the tumors were checked by PET/CT surveillance. Then, mice were injected intraperitoneally with honokiol (5 mg/kg/twice per week) for 28 days. The effect of honokiol on peritoneal dissemination was evaluated by PET/CT. A statement/picture of the metastasis situation was described in Figure 1. Then, the mice were sacrificed under anesthesia (pentobarbital) and examined macroscopically for the presence of peritoneal metastasis. The tumors were excised, cut into blocks, fixed in 10% formalin, and embedded in paraffin blocks or snap-frozen in liquid nitrogen.

### Positron Emission Tomography/Computed Tomography (PET/CT) Scanning

After at least 6 h of fasting, the mice were given a 7.4 MBq (0.2 mCi) dose of ^18^F-FDG orally before flushing with 1 mL of water. The mice were anesthetized with an isoflurane vaporizer before each scan. Experiments for small animal imaging were performed with a combined PET/CT scanner (Discovery ST; GE Medical Systems). A multidetector row helical CT scanner was used. Technical parameters used in the CT portion of PET/CT were as follows: CT scan type with a 0.5-second full helical scan, a detector row configuration of 16×1.25 mm, an interval space of 2.75 mm, a slice thickness of 1.25 mm, a pitch of 1.75∶1 (high quality mode), a speed of 17.5 mm per rotation, a large field-of-view (FOV), voltage of 120 kVp and current of 200 mA. Technical parameters used for the PET portion of PET/CT were as follows: 10.0 min in each bed, the FOV chosen for imaging reconstruction was 20 cm and the PET resolution was about 4.5 mm full width at half maximum (FWHM). The reconstructive parameters were type 3D iteration as 21 subsets and 2 iterations. To evaluate the quantification ability of the PET/CT scanner in small animal imaging, a region of interest (ROI) was placed on smaller FOV transaxial PET images to completely surround the areas of FDG uptake in the observed tissues while avoiding nearby tissues. The mean of each pixel’s activity value within each ROI was recorded and expressed as Bq/ml.

### Chick Chorioallantoic Membrane Assay (*CAM* Assay)

To investigate the *in vivo* angiogenic activity, a modified chick whole animal *CAM* assay was carried out as described previously [43]. Fertilized chick eggs were incubated in a continuously humidified egg breeder at 37°C. At 3 days of incubation, chick embryos were transferred to a culture dish. Twenty µL of honokiol entrapped in type I collagen mixture was applied onto coating disks and polymerized by warming. The disks were loaded onto the *CAM* of 6-day-old embryos. After 24-h incubation, the area around the loaded disk was photographed with a Nikon digital camera, and the number of newly formed vessels was counted by two observers in a double-blind manner. Assays for each test sample were carried out using 10–13 eggs.

### Matrigel Plug Assay

Nude mice or C57BL/6 mice were injected subcutaneously with 0.6 mL of matrigel containing the indicated amount of capsaicin, 100 ng of VEGF, and 10 units of heparin. The injected matrigel rapidly formed a single, solid gel plug. After 7–21 days, the skin of the mouse was easily pulled back to expose the matrigel plug, which remained intact. Hemoglobin was measured using the Drabkin method and a Drabkin reagent kit 525 (Sigma, St Louis, MO) for the quantification of blood vessel formation. The concentration of hemoglobin was calculated from a known amount of hemoglobin assayed in parallel. The images were photographed with a Nikon digital camera, and the number of newly formed micro-vessels was counted.

### *Ex Vivo* Aortic Ring Assay

Aortas were isolated from 6-week-old Sprague-Dawley rats. Plates (48-well) were coated with 120 µL of matrigel. Aortas isolated from mice were cleaned of periadventitial fat and connective tissues, and cut into ∼1-mm-long to 1.5-mm-long rings. After being rinsed five times with endothelial cell–based medium, the aortas were placed on the matrigel-covered wells and covered with another 100 µL of matrigel. Honokiol or vehicle was added to the wells in a final volume of 250 µL of medium. Cultures were incubated and the media were replaced every second day over the course of the 8–10 days of experiments. Visual counts of micro-vessel outgrowths from replicate explant cultures (n≥6) were done under bright-field microscopy following an established protocol The assay was scored from 0 (least positive) to 5 (most positive) in a double-blinded manner. Each data point was assayed in sextuplets. Experiments were performed at least twice, and micro-vessel counts in treated and control cultures were analyzed.

### Endothelial Cell Tube Formation

Matrigel was thawed at 4°C overnight, and each well of prechilled 24-well plates was coated with 100 µL of matrigel and incubated at 37°C for 45 min. HUVECs (4×10^4^ cells) were added to 1 mL of ECGM with various concentrations of honokiol. After 12 h of incubation at 37°C, 5% CO_2_, endothelial cell tube formation was assessed with a Nikon inverted microscope with attached digital camera. Tubular structures were quantified by manual counting of low power fields (50×), and the inhibition percentage was expressed using untreated wells as 100%.

### Western Blot Analysis and Immunoprecipitation

Protein levels were analyzed by Western blot as described previously [41]. Antibodies against calpain, SHP-1, SHP-2 (Santa Cruz Biotechnology), STAT-3, p-STAT-3 (Tyr705 and Ser727) (New England BioLabs), and β-actin (Sigma) were used. For immunoprecipitation, proteins (500 µg) were incubated with specific antibodies and immobilized onto protein A-Sepharose beads.

### Immunofluorescence and Laser Scanning Confocal Microscopy

The immunofluorescence of phosphorylated STAT3 expression in gastric cancer cells and HUVECs was determined by a laser scanning confocal microscope [16]. Images were background-subtracted and merged using the Confocal Assistant MetaMorph software program, and processed with Adobe Photoshop software.

### RNA Interference and Transfection Assays

The delivery of siRNA pools into cells was performed using Lipofectin (Invitrogen). The pooled siRNA duplexes were dissolved in buffer (20 mM KCl, 6 mM HEPES, pH 7.5, and 0.2 mM MgCl_2_) to a concentration of 20 µM. Cell transfection was carried out for 24 h at a final siRNA concentration of 100 pM. Control cells were mock-transfected without siRNA, and all experimental results were confirmed using scrambled siRNA. The commercial siRNA products for human SHP-1, calpain II, and scrambled control were used and purchased from Santa Cruz Biotechnology (SHP-1 siRNA, sc-29478; Calpain II siRNA, sc-41459; Control siRNA, sc-37007).

### Immunohistochemistry

The expressions of SHP-1 and p-STAT-3 proteins in mouse solid tumors were examined by immunohistochemistry. All tumors were fixed in 10% buffered formalin immediately after the animals were sacrificed. After pressure cooker pretreatment in citrate buffer (pH 6.0) for 30 min, 5 mm sections of the tumors were incubated at room temperature for 1 h with a monoclonal antibody for SHP-1, phospho-VEGFR-2 (1∶250, Santa Cruz Biotechnology), and CML (1∶250, Trans Genic)., (phospho-STAT-3 (dilution: 1∶250, Cell Signaling Technology). After incubation with an appropriate primary antibody enhancer, the slides were incubated with HRP polymer (Laboratory Vision Corporation, Fremont, CA, USA). Reaction products were visualized by immersing the slides in peroxidase-compatible chromogen. Finally, the slides were counterstained with hemalaun.

### Electrophoretic Mobility Shift Assay

The electrophoretic mobility shift assay was performed as described previously [16]. The consensus oligonucleotide probes were end-labeled with [γ-^32^P]ATP according to the manufacturer’s recommendations. For the binding reaction, 2 ng of the labeled oligonucleotide (approximately 20,000 cpm) and 2 µg of poly dIdC (Amersham Pharmacia Biotech) carrier were incubated with 2 µg of nuclear protein in a binding buffer (10 mM HEPES, 60 mM KCl, 1 mM DTT, 1 mM EDTA, 7% glycerol, pH 7.6) for 30 min at room temperature. Protein-DNA complexes were separated by electrophoresis on a nondenaturing 6% polyacrylamide gel and visualized by autoradiography. For competition experiments, a 100-fold excess of the unlabeled oligonucleotides was added 15 min before incubation of nuclear extracts with the end-labeled oligonucleotides.

### SHP-1 Phosphatase Activity Assay

Five hundred µg of supernatant protein were incubated with 2 µg of antiphosphorylated SHP-1 antibody or IgG at 4°C for 2 h, followed by incubation with protein A/G PLUS for 1 h. The precipitates were used to measure SHP-1 phosphatase activity. Phosphatase activities of immunoprecipitated SHP-1 were analyzed using a *p*NPP protein phosphatase assay kit (AnaSpec) according to the manufacturer’s instructions. The absorbance at 405 nm was determined by a microplate reader.

### Transmission Electron Microscopy

The ER structure in cells was observed by transmission electron microscopy as described previously [44]. Cells were treated with or without drugs for 18 h. Cells were harvested and then fixed with 4% glutaraldehyde and 2.5% paraformaldehyde dissolved in 0.1 M of sodium cacodylate. The sections were prepared with an LKB Ultracut microtome (Leica, Deerfield, IL) and then stained with uranyl acetate and lead citrate in an LKB Ultrostainer. The sections were examined in a JEM 1200 EX transmission electron microscope (JEOL, Peabody, MA) at an accelerating voltage of 80 kV.

### Analysis of VEGF Generation

VEGF concentrations were measured with a human VEGF ELISA kit (R&D Systems).

### Statistics

The values given in this study are presented as mean ± SEM. Analyses of variance followed by a Fisher’s least significant difference test were performed for all data. *P* values of less than 0.05 were considered statistically significant.
